# Editorial: Community series in immunotherapy with checkpoint inhibitors for non-small cell lung cancer, colon cancer, and esophageal cancer, volume II

**DOI:** 10.3389/fimmu.2023.1222675

**Published:** 2023-07-03

**Authors:** Yueyi Li, Yusha Wang, Jinsheng Gao, Keqin Tan, Benjamin Frey, Udo S. Gaipl, Hubing Shi, Xuelei Ma

**Affiliations:** ^1^ Department of Biotherapy, Cancer Center and State Key Laboratory of Biotherapy, West China Hospital, Sichuan University, Chengdu, Sichuan, China; ^2^ People’s Hospital of Yilong County, Nanchong, Sichuan, China; ^3^ Translational Radiobiology, Universitätsklinikum Erlangen, Erlangen, Germany; ^4^ Laboratory of Integrative Medicine, Clinical Research Center for Breast, State Key Laboratory of Biotherapy, West China Hospital, Sichuan University and Collaborative Innovation Center, Chengdu, Sichuan, China

**Keywords:** mmune checkpoint inhibitors (ICIs), non-small cell lung cancer (NSCLC), colorectal cancer, esophageal cancer (EC), editorial

## Introduction

Immunotherapy with checkpoint inhibitors has revolutionized the treatment of various types of cancer. Non-small cell lung cancer, colorectal cancer, and esophageal cancer are among the most common cancers globally, but treatment options for advanced stages have historically been limited. However, with the advent of checkpoint inhibitors, significant progress has been made in improving patient outcomes and extending survival rates (1). This Research Topic brings together 18 studies from researchers in different countries, showcasing the latest advances in immunotherapy for these three types of cancer. The inclusion of varied perspectives and international insights enhances the depth and breadth of knowledge in the field, ultimately benefiting patients around the world.

## Application of ICIs in NSCLC

In the realm of lung cancer treatment, immune checkpoint inhibitors (ICIs) have emerged as a game-changing approach. These inhibitors, such as programmed cell death-1 (PD-1) inhibitors, programmed cell death ligand 1 (PD-L1) inhibitors, and cytotoxic T-lymphocyte antigen 4 (CTLA-4) inhibitors, have demonstrated remarkable clinical advancements, predicting outcomes and improved patient tolerability. Liu Y. et al. presented the research trends on lung cancer, emphasizing the importance of immunotherapy. In patients with advanced NSCLC, the combination of PD-1/PD-L1 inhibitors with anti-angiogenic drugs, with or without chemotherapy, was found to be superior to PD-1/PD-L1 inhibitors plus chemotherapy as a second- or later-line treatment (Chen S. et al.). Moreover, in the prediction of prognosis, a decreased monocyte-to-lymphocyte ratio (MLR) has been associated with a high objective response rate and long progression-free survival, indicating its potential predictive value for first-line PD-1 inhibitor combination chemotherapy in stage IIIB-IV NSCLC patients, as well as for all PD-L1-expressing populations. The changes in the neutrophil-to-lymphocyte ratio (NLR) and platelet-to-lymphocyte ratio (PLR) may supplement the prediction of prognosis (Zheng et al.). Furthermore, Lin M. et al. reviewed the outcomes of patients with lung cancer (LC) with chronic obstructive pulmonary disease (COPD) after anti-PD-1/PD-L1 treatment and found that LC patients with COPD would benefit more from immunotherapy. In addition, PD-L1 expression has been found to be a predictor of tumor response, and PD-1/PD-L1 inhibitors have demonstrated good efficacy in treating patients with pulmonary lymphoepithelioma-like carcinoma (LELC) in retrospective cohort studies, which warrants further investigation in prospective studies as a frontline treatment option (Zhou N. et al.). Furthermore, an innovative approach has shown promise for patients with specific biomarkers and distinct tumor microenvironment (TME) characteristics. The expression of ALK/EGFR can be useful in the treatment and prognosis of non-small cell lung cancer. Patients with ALK-positive and EGFR/KRAS-positive NSCLC have been shown to exhibit an immunosuppressive TME, with high expression of PD-L1 and CTLA4 as poor prognostic factors in advanced ALK-rearranged NSCLC patients treated with ALK-TKI (Zhang B. et al.). Lin J. et al. have identified a possible cancer vaccine produced by the EGFR L858R neoantigen in non-small cell lung cancer patients with HLA A*33:03, which may serve as an effective remedy for the EGFR L858R subgroup after failed targeted therapy or ICI treatment. Moreover, the basement membranes- tumor immune microenvironment classifier has demonstrated its prognostic value in multiple cohorts. This can guide clinical decision-making and therapeutic strategies for patients with NSCLC (Lin J. et al.).

## Application of ICIs in colorectal cancer

In recent years, immunotherapy has made great progress in colorectal cancer. For metastatic colorectal cancer, immune checkpoint blockade therapy has been approved for the treatment of patients with mismatch-repair-deficient (dMMR) and who have high levels of microsatellite instability (MSI-H). As for early-stage colon cancer with dMMR/MSI-H, neoadjuvant immunotherapy seems to be a promising treatment. Zhou L. et al. comprehensively analyzed neoadjuvant immunotherapy and found that this type of immunotherapy could increase pCRs and MPR rates for the dMMR/MSI-H group of non-metastatic colorectal cancer (Zhou L. et al.). Furthermore, given high ORR and pCR rates, low incidence of irAE and srAE, PD-1 inhibitors have shown great efficiency as neoadjuvant mono-immunotherapy for early-stage colorectal cancer with dMMR/MSI-H (Zhang X. et al.). Predictive biomarkers that can indicate immune infiltration and immunotherapy response contribute to guiding immunotherapy for colon cancer. Hou et al. comprehensively summarized reported predictive biomarkers for colon cancer immunotherapy and discussed the prospects for technological change in colon cancer immunotherapy biomarker development (Hou et al.). Novel biomarker ALOX12 is proven to predict bevacizumab response, immunotherapy effect, and prognosis of colorectal cancer (Weng et al.). It is well known that various ICIs have different immune-related adverse events (irAEs), which may involve many organs, such as the lung, liver, skin, kidney, digestive system, or endocrine system. Wang S. et al. reported a patient with locally advanced colorectal cancer who developed tislelizumab-induced multiple organ irAEs, and treatments including intravenous immunoglobulins (IVIGs) and corticosteroids improved these symptoms (Wang S. et al.). In addition, ICIs with nanotechnology have shown to be effective to avoid undesired side effects, unsatisfactory response rates, tumor metastasis, and drug resistance (Liu Z. et al.).

## Application of ICIs in esophageal cancer

Esophageal cancer (EC) is a malignant disease and remains one of the leading causes of death. Immunotherapies such as immune checkpoint inhibitors, cancer vaccines, and adoptive cell therapy are effective for patients with EC. Wang H. et al. summarized and systematically analyzed immunotherapy-based combination therapies for EC (Wang H. et al.). As mentioned above, a strong immune response may lead to more serious and multi-system irAE. However, it has been found that patients with irAEs showed markedly better efficacy in ORR, DCR, PFS, and OS in advanced EC (Qin et al.). In summary, the occurrence of adverse effects may indicate that patients may benefit from immunotherapy, but serious adverse effects should be avoided.

This Research Topic provides a series of new research findings and insights that highlight the potential of immunotherapy and checkpoint inhibitors in non-small-cell lung carcinoma, colorectal, and esophageal cancers. Zhao et al. and Botticelli et al. provided a deeper understanding of immunotherapy for cancers by providing up-to-date results, exploring new therapeutic options, and evaluating existing therapies. Several studies included in this Research Topic have shown that checkpoint inhibitors have emerged as an effective treatment option to improve survival in some patients. For example, some studies have shown that PD-1 and PD-L1 antibody combination therapy in patients with non-small-cell lung carcinoma increases survival and improves response to treatment. Overall, these articles provide an in-depth insight into the role of immunotherapy and checkpoint inhibitors in these cancer types, which can help physicians, researchers, and patients better understand these treatments and guide future research direction and clinical practice. The significance and contribution of these articles are that they advance our understanding of the role of immunotherapy and checkpoint inhibitors in cancer therapy and provide new ideas and directions for future research and treatment.

In conclusion, this Research Topic represents a significant milestone in the field of cancer immunotherapy. It showcases groundbreaking research, novel treatment strategies, and valuable insights into the mechanisms of response and resistance to checkpoint inhibitors. By addressing clinical challenges and providing evidence-based recommendations, this article Research Topic aims to improve patient outcomes and shape the future of immunotherapeutic approaches for these specific cancer types.

## Author contributions

Literature review and data collection were performed by YL and YW. The first draft of the manuscript was written by YL, JG, and KT. The final version of the editorial was written by UG, BF, HS, and XM. All authors read and approved the final manuscript.
